# Downhill running increases markers of muscle damage and impairs the maximal voluntary force production as well as the late phase of the rate of voluntary force development

**DOI:** 10.1007/s00421-023-05412-z

**Published:** 2024-01-10

**Authors:** Giuseppe Coratella, Giorgio Varesco, Vianney Rozand, Benjamin Cuinet, Veronica Sansoni, Giovanni Lombardi, Gianluca Vernillo, Laurent Mourot

**Affiliations:** 1 Department of Biomedical Sciences for Health, Università Degli Studi Di Milano, Milan, Italy; 2 Université Jean Monnet Saint-Etienne, Inter-University Laboratory of Human Movement Biology, 42023 Saint-Etienne, France; 3 Laboratory Movement—Interactions—Performance, MIP Lab, UR 4334, Nantes Université, F-44000 Nantes, France; 4 Prognostic Factors and Regulatory Factors of Cardiac and Vascular Pathologies (EA3920), Exercise Performance Health Innovation (EPHI) Platform, University of Franche-Comté, Besançon, France; 5 Laboratory of Experimental Biochemistry and Molecular Biology, IRCCS Instituto Ortopedico Galeazzi, 20157 Milan, Italy; 6 Department of Athletics, Strength and Conditioning, Poznań University of Physical Education, 61-871 Poznań, Poland

**Keywords:** Creatine kinase, Delayed onset muscle soreness, Eccentric, Maximum voluntary contraction, Muscle swelling, Musculoskeletal, Quadriceps, Trail running

## Abstract

**Purpose:**

To examined the time-course of the early and late phase of the rate of voluntary force development (RVFD) and muscle damage markers after downhill running.

**Methods:**

Ten recreational runners performed a 30-min downhill run at 10 km h^−1^ and −20% (−11.3°) on a motorized treadmill. At baseline and each day up to 4 days RVFD, knee extensors maximum voluntary isometric force (MVIC), serum creatine kinase (CK) concentration, quadriceps swelling, and soreness were assessed. The early (0–50 ms) and late (100–200 ms) phase of the RVFD, as well as the force developed at 50 and 200 ms, were also determined.

**Results:**

MVIC showed moderate decrements (p < 0.05) and recovered after 4 days (p > 0.05). Force at 50 ms and the early phase were not impaired (p > 0.05). Conversely, force at 200 ms and the late phase showed moderate decrements (p < 0.05) and recovered after 3 and 4 days, respectively (p > 0.05). CK concentration, quadriceps swelling, and soreness increased (p < 0.05) were overall fully resolved after 4 days (p > 0.05).

**Conclusion:**

Downhill running affected the knee extensors RVFD late but not early phase. The RVFD late phase may be used as an additional marker of muscle damage in trail running.

## Introduction

Performing eccentric exercise is known to induce symptoms of muscle damage (Clarkson and Hubal 2002). Decreased force, as well as increased muscle soreness, swelling and/or muscle protein activity are among the most common indicators used to monitor the reparation process taking place the days following the eccentric session (Nosaka and Clarkson 1995). However, they manifest with an unsynchronized time-course after the eccentric exercise. Interestingly, eccentric exercise can be performed in different modalities, and muscle damage has been investigated using dynamic constant external load or isokinetic device (Coratella and Bertinato 2015), flywheel instruments (Coratella et al. 2016), eccentric cycling (Peñailillo et al. 2015; Mavropalias et al. 2020), and downhill running (Byrnes et al. 1985; Khassetarash et al. 2021). Particularly, downhill running places unique biomechanical, neuromuscular, and physiological challenges on the human body (Giandolini et al. 2016; Vernillo et al. 2017, 2020; Bontemps et al. 2020; Khassetarash et al. 2020, 2021). Therefore, it has raised a scientific interest given the increasing participation in trail and ultratrail running (Hoffman et al. 2010), whose downhill running seems to be a crucial aspect of the performance (Bontemps et al. 2020). Using downhill running as an eccentric exercise model could (i) inform aspects of training preparation in trail and ultratrail events, and (ii) minimize the risk of overuse injuries associated with greater impact loading and muscle damage.

While maximal force is generally considered as the gold standard to evaluate global changes in neuromuscular function, it was recently suggested that this evaluation may be incomplete, and the rise in muscle force should also be considered for a more comprehensive evaluation (Maffiuletti et al. 2016). Although instantaneous force values at specific time points could provide further insights, the rate of voluntary force development (RVFD) has been more recently adopted for a more comprehensive evaluation of a task-induced neuromuscular changes (D’Emanuele et al. 2021). The RVFD denotes the ability of the neuromuscular system to rapidly increase force over time (e.g., usually in the first 200–250 ms) (Maffiuletti et al. 2016). Specifically, the slope from the first 50–100 ms of the force–time curve has been described as the early phase, and mainly depends on neural aspects such as the firing of the motor units for the muscles involved in the task (Del Vecchio et al. 2019). In contrast, the slope from 100 to 250 ms has been described as the late phase, and depends on a series of structural factors such as muscle morphology (Aagaard et al. 2002), muscle architecture (Coratella et al. 2020), and tendon stiffness (Maffiuletti et al. 2016).

Muscle damage is initiated by a disruption of the sarcomeres and an impairment in the excitation–contraction coupling (Proske and Morgan 2001). Both mechanisms may have implications on the RVFD, specifically on its late phase since they refer to the muscle structure instead of the neural component. After an eccentric cycling bout for 30 min at 60% of maximal concentric power output, Peñailillo et al. (2015) showed that RVFD can be considered an indirect marker of eccentric exercise-induced muscle damage. However, several physiological and biomechanical differences between running and cycling exist (Millet et al. 2009), so whether or not the RVFD may present the same behaviour after downhill running remains to be investigated. In addition, Peñailillo et al. (2015) stopped the examination at 48 h after the eccentric protocol, while it is known that muscle damage may last much further (Clarkson and Hubal 2002). Therefore, the present study aimed to examine the time-course alterations of the early and late phase of RVFD induced by a session of downhill running, together with other traditional muscle damage markers. This information could be of practical significance in trail running and following prolonged duration races wherein cumulative downhill eccentric loading is high.

## Materials and methods

### Participants

Ten healthy male recreational runners volunteered to participate in the present study (age: 22 ± 3 years, height: 181 ± 8 cm, body mass: 79 ± 8 kg) (Peñailillo et al. 2015). Participants were considered “recreational runners” if they ran less than 4 days per week (Kuru 2016; Clermont et al. 2020). Prior to their inclusion in this study, the participants were screened for the following exclusion criteria: smoking, current medication or drug consumption, and presence of apparent cardiovascular, metabolic, neurologic, or musculoskeletal disease. Furthermore, participants were excluded if they practiced regular eccentric-based resistance training within a 6-month period prior to the start date of this study, as well as prolonged (> 20 min) and repeated (more than two times per week) downhill running practice. Participants were also excluded if they were already familiarized with other types of eccentric exercises (e.g., strength training) prior to this study. They were also instructed to avoid (i) aspirin, ibuprofen, or other anti-inflammatory drugs; (ii) the consumption of caffeine and/or alcohol on the day of the experiment; and (iii) any strenuous exercise for the entire duration of the investigation. All procedures were approved by the local ethics committee and this study conformed to the standards set by the Declaration of Helsinki (1965 and further modifications), except for registration in a database.

### Experimental protocol

To investigate the duration of the downhill-induced muscle damage, the participants were tested at baseline (PRE) and 24 (POST24), 48 (POST48), 72 (POST72) and 96 (POST96) hours after the downhill bout. Participants first came for two familiarization sessions (separated by between 3 and 7 days), and again 1 week after the second familiarisation session for the experimental session. During the first familiarisation session, participants were familiarised with the procedures to identify the maximum voluntary isometric contraction (MVIC) and the RVFD. During the second familiarisation session, participants performed a single 5-min downhill run, replicating the experimental settings on the same treadmill used for the experimental session. Both the baseline assessments and the downhill running session were conducted on the same days. Room temperature and humidity were similar across sessions (21 °C, 45% RH). For each assessment session, the order was fixed as follows: venous blood samples, muscle soreness, muscle swelling, MVIC and RVFD (Chapman et al. 2008).

### Maximum voluntary isometric contraction

Unilateral right limb knee extensors MVIC was assessed with the participants seated with the hip and knee angles at 90° on a customized seat with a calibrated force transducer (Legcontrol, Mtraining, Ecole Valentin, France). In line with a previous investigation (Varesco et al. 2022), the lever arm of the dynamometer was adjusted to firmly attach the leg 3 cm above the medial malleoli with two non-compliant belts and a belt strapped over the waist was used to minimize extraneous movements of the upper body. Passive resting force was subtracted from the signal so that the baseline was set at 0 N (Varesco et al. 2022). Force signals were collected without analogical filters at a frequency of 2 kHz by PowerLab System (16/30-ML880/P, AD Instruments, Bellavista, Australia). The MVIC assessment was preceded by a standardized warm up consisting of 20 × 2-s knee extensions separated by 10 s each, and the participants were instructed to gradually increase the force up to the maximum volitional force (Coratella et al. 2020). After 5 min, two 4-s MVIC attempts were performed, separated by 5 min of passive rest. At baseline, if the difference between the two MVICs was > 5%, further trials were performed until the difference between two consecutive trials was < 5%. The participants were instructed to “push as hard as possible” and strong standardized encouragements were provided by the operators at each attempt. The maximum value was then retained for further analysis.

### Rate of voluntary force development

**Fig. 1:**
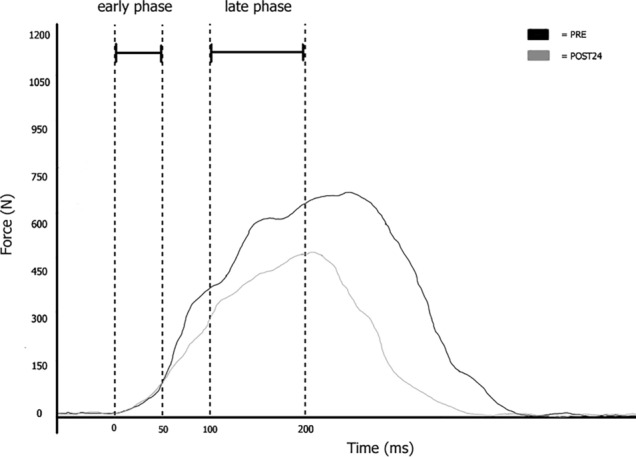
Representative participant’s typical traces of the rate of voluntary force development (RVFD) at different time intervals from the onset before (PRE) and 24 h after (POST24) the downhill bout

The RVFD was assessed after 10 min of passive rest on the same customized seat and with the participants in the same position as for the MVIC. The participants were requested to “push as fast as possible” during eight impulsive unilateral right limb isometric knee extensions (Varesco et al. 2019). In case of a countermovement (determined by a force drop of 2 N below the baseline right before the impulsive contraction) or pre-tension (determined by a force level ≥ 2 N above the baseline right before the impulsive contraction) the contraction was repeated. The impulsive contractions were also repeated if the force level was < 70% of the MVIC that preceded the series of impulsive contractions (Varesco et al. 2022). All testing protocols were performed with real-time visual feedback. For each impulsive contraction, we computed the first derivative of the force–time curve, and retrieved the peak value as the point of maximal acceleration during the contraction. We then extracted and average value from data 5 ms before to 5 ms after this data point to compute peak RVFD value (Varesco et al. 2019). The onset of the voluntary force development was automatically defined as the point at which force exceeded the average resting baseline by ~ 2 N. The onset was also checked visually by an experienced operator blinded to the condition. The force was measured at 50 and 200 ms and the RVFD was calculated fitting a linear model over the 0–50 ms (RVFD_0–50_) and 100–200 ms (RVFD_100–200_) time-windows data points and extracting the slope of the model (Fig. 1). All data were analysed offline using Labchart 8 Software (ADInstruments, Bella Vista, Australia) and then retained for further analysis.

### Serum creatine kinase

Venous blood samples (~ 5 mL) were collected by standard venipuncture of the antecubital vein in SST II Advance Vacutainer^®^ and in K_2_EDTA tubes (Becton, Dickinson & Co., Franklin Lakes, NJ, USA). Then it was immediately centrifuged at 3000 × g (15 min, 4 °C) and serum and plasma was aliquoted and stored at −80 °C until assayed. Serum concentration of muscle creatine kinase isoform (CKM), including skeletal (CK-MM) and cardiac (CK-MB) muscle isoenzymes, was measured by a SimpleStep ELISA^®^ (Abcam, Cambridge, UK). Sensitivity, intra-assay (CVw), and inter-assay (CVb) variations were 270 pg/mL 2.2% and 7.4%, respectively.

### Muscle swelling

To evaluate muscle swelling, the right thigh circumference was measured at 50% of the distance between the iliac spine and the patella using a seamstress meter with the participant seated, knee at 90° flexion (with 0° being full extension) and the lower limbs relaxed. To ensure consistency, the site was marked with a semipermanent ink. Three measurements were taken, and the mean was then retained for further analysis.

### Muscle soreness

Muscle soreness was investigated as pressure pain threshold using an algometer (Pain Diagnostics and Thermography, New York, USA) on the right vastus medialis at 50% of the distance between the anterior superior iliac spine and the patella. The probe was placed perpendicular to the site and the investigator gradually applied force until the participant reported pain (Fischer 1987). Three measurements were performed, and the mean was then retained for further analysis.

### Downhill running

The participants performed a 30-min downhill run at 10 km h^−1^and with a slope of −20% (−11.3°) on a motorized treadmill (Medic 2855, Genin Medical, La Roque-d’Anthéron, France) (Varesco et al. 2022). We used treadmill running given the similarity in running biomechanics observed with the downhill overground running (Firminger et al. 2018).

### Statistical analysis

Results are given as means ± standard deviations. The normality of distribution was verified using the Shapiro–Wilk normality test. To test differences between PRE and POST24/48/72/96, a longitudinal analysis was performed using generalized estimating equations (GEE; i.e., GEE under ‘generalized linear model’ procedure in SPSS v. 28) to take into account the correlated nature of observations within each participant (i.e., within-participant measurements) (Liang and Zeger 1986). If a significant main effect for time was observed, Bonferroni’s test was used for post hoc analysis. As a measure of effect size, Cohen’s d (d) was calculated and interpreted as follows: < 0.19 = trivial, 0.20–0.59 = small, 0.60–1.19 = moderate, 1.20–1.99 = large, > 2.00 = very large (Hopkins et al. 2009). The statistical analyses were conducted using IBM™ SPSS™ Statistics (version 28.0.0; IBM Corp., Somers, New York, NY) with the criterion α level set to 0.05.

## Results

### Rate of voluntary force development

**Fig. 2:**
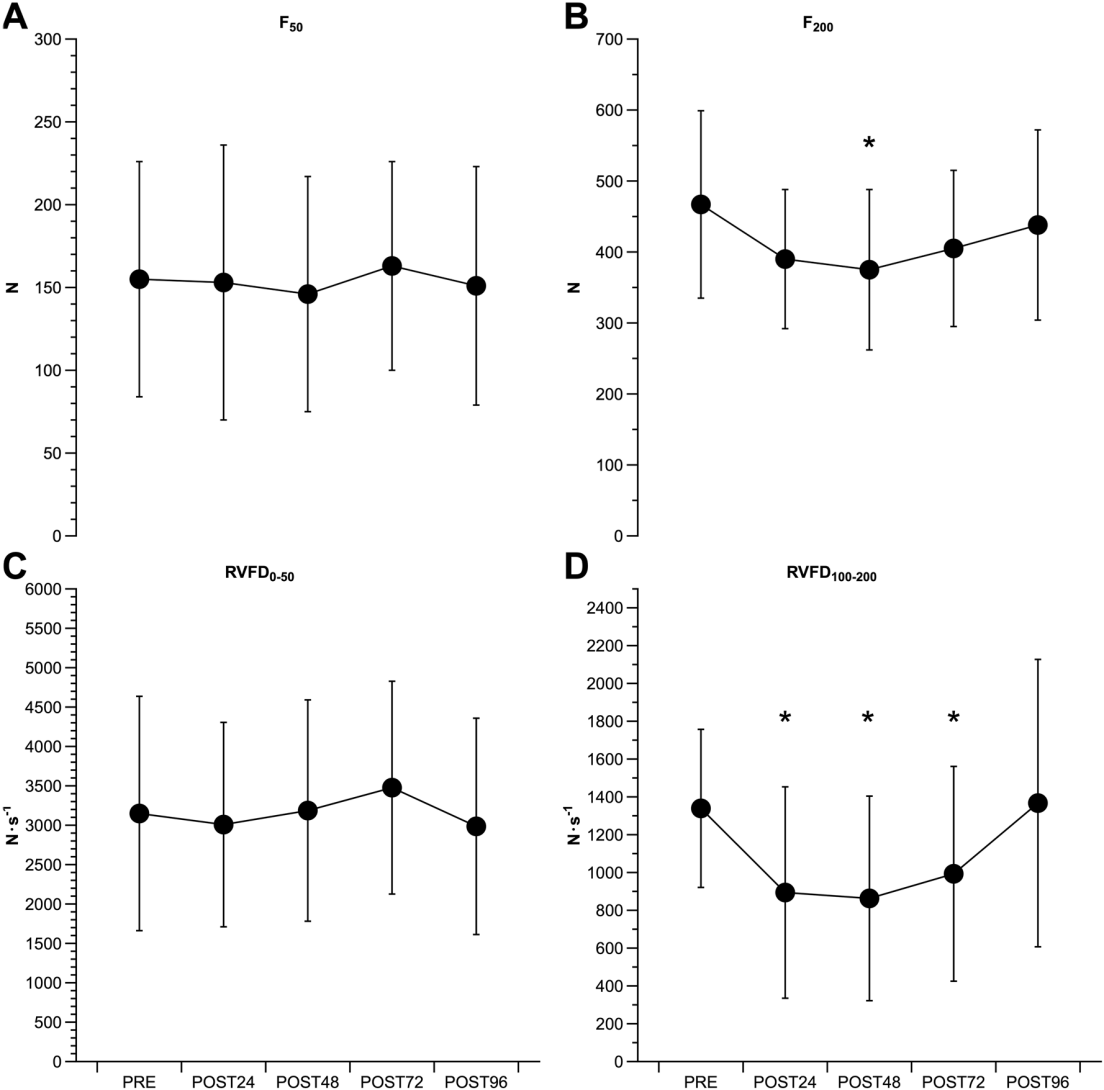
The time-course of the force exerted at 50 ms (**A**) and 200 ms (**B**), and of the early (**C**) and the late phase (**D**) of the knee extensors RVFD. ^*^p < 0.05 vs PRE

Force at 50 ms did not show a time effect [_w_χ^2^ (4) = 2.0, p = 0.732] (Fig. 2A). Force at 200 ms showed a time effect [_w_χ^2^ (4) = 16.7, p = 0.002] (Fig. 2B). Force at 200 ms was only lower at POST48 compared to PRE (467 ± 132 N vs 375 ± 113 N, 83 ± 20% of PRE values, p = 0.045, d = 0.75). RVFD_0–50_ showed a time effect [_w_χ^2^ (4) = 9.8, p = 0.044]. However, when PRE was compared to the other time points, no difference was detected (all p = 1.000) (Fig. 2C). RVFD_100–200_ showed a time effect [_w_χ^2^ (4) = 42.2, p < 0.001]. Compared to PRE (1339 ± 418 N s^−1^), RFD100–200 was lower at POST24 (894 ± 559 N s^−1^, 63 ± 28% of PRE values, p < 0.001, d = 0.90), and remained lower until POST72 (993 ± 568 N s^−1^, 74 ± 31% of PRE values, p = 0.030, d = 0.69) (Fig. 2D).

### Maximum voluntary isometric contraction

**Fig. 3:**
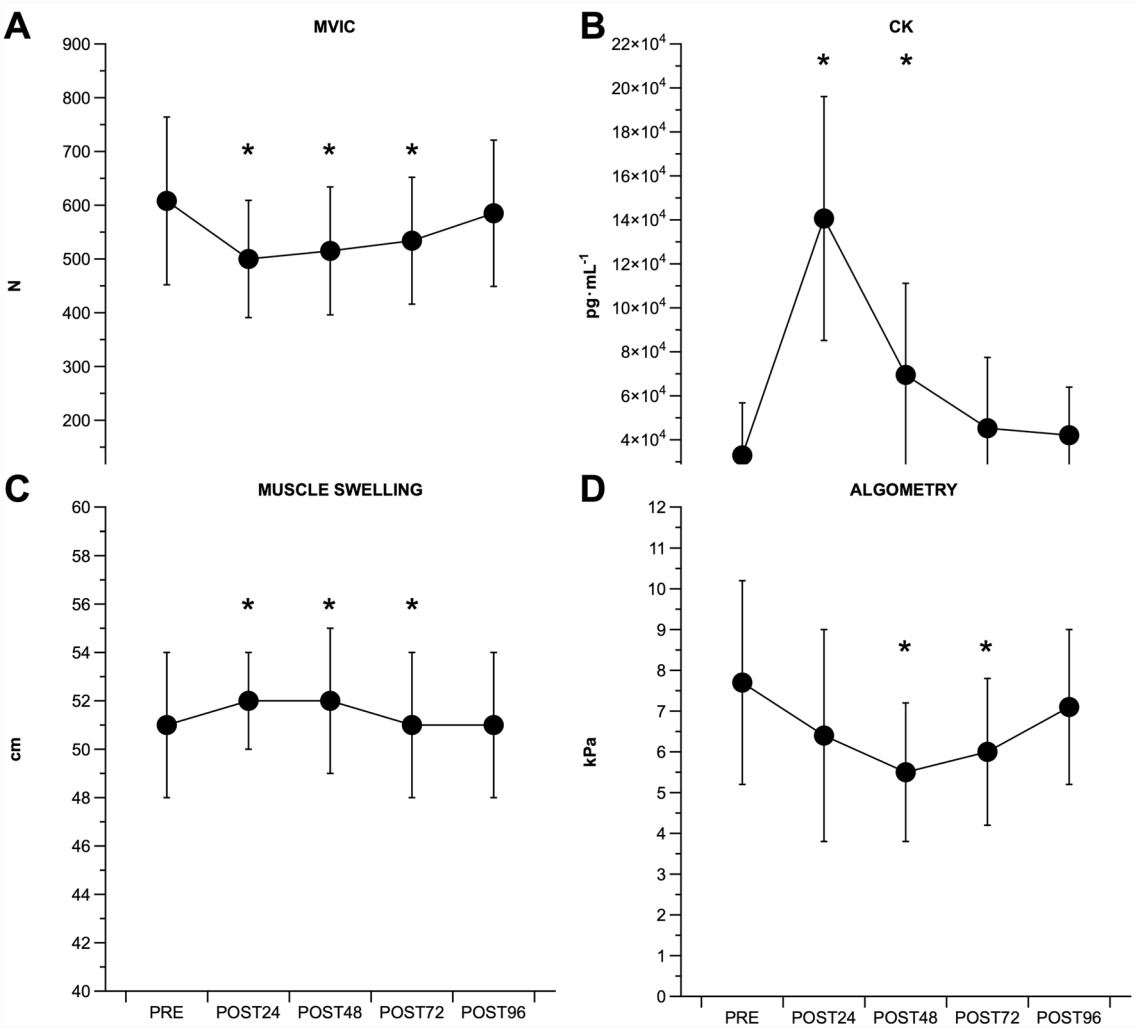
The time-course of the maximum voluntary isometric contraction (MVIC) (**A**), serum creatine kinase (CK) concentration (**B**), muscle swelling (**C**) and soreness (algometry) (**D**). ^*^p < 0.05 vs PRE

MVIC force showed a time effect [_w_χ^2^ (4) = 17.7, p = 0.001]. Compared to PRE (608 ± 156 N), MVIC force was lower at POST24 (500 ± 109 N, 84 ± 13% of PRE values, p = 0.002, d = 0.80), and remained lower until POST72 (534 ± 118 N, 90 ± 13% of PRE values, p = 0.026, d = 0.53) (Fig. 3A).

### Serum creatine kinase

Creatine kinase (CK) showed a time effect [_w_χ^2^ (4) = 46.2, p < 0.001]. Compared to PRE (32,923 ± 23,874 pg mL^−1^), CK was higher at POST24 (147,273 ± 55,447 pg mL^−1^, 613 ± 394% of PRE values, p < 0.001, d = 2.68), and POST48 (69,495 ± 41,670 pg mL^−1^, 232 ± 118% of PRE values, p < 0.001, d = 1.08) (Fig. 3B).

### Muscle swelling

Muscle swelling showed a time effect [_w_χ^2^ (4) = 55.0, p < 0.001]. Compared to PRE (51 ± 3 cm), muscle swelling was higher at POST24 (52 ± 2 cm, 102 ± 1% of PRE values, p < 0.001, d = 0.43), and remained higher until POST72 (51 ± 3 cm, 102 ± 2% of PRE values, p = 0.005, d = 0.32) (Fig. 3C).

### Muscle soreness

Muscle soreness showed a time effect [_w_χ^2^ (4) = 19.0, p < 0.001]. Compared to PRE (7.7 ± 2.5 kPa), muscle soreness decreased at POST48 (5.5 ± 1.7 kPa, 77 ± 27% of PRE values, p = 0.007, d = 1.03), and POST72 (6.0 ± 1.8 kPa, 81 ± 18% of PRE values, p = 0.005, d = 0.78) (Fig. 3D).

## Discussion

The present study was conceived to determine whether RVFD is sensitive to the muscle damage following downhill running. The effectiveness of the downhill running bout in inducing muscle damage was confirmed by a reduction in the MVIC, as well as by an increase in muscle swelling, CK concentration, and muscle soreness. Overall, our results showed that a downhill running session impaired the RVFD_100–200_ for 3 days after the bout, while the RVFD_0–50_ was not affected. Taken together, the present results suggest that the late phase of RVFD appears sensitive to the muscle damage induced after an eccentric-biased downhill running, while the early phase does not.

We found moderate decrements for the RVFD_100–200_, while the RVFD_0–50_ was not affected by a bout of downhill running. While the early phase is mostly determined by neural factors, the late phase of RVFD is mainly based on muscular determinants (Maffiuletti et al. 2016). Among others, the type-II fibres have been pointed as more advantageous for producing faster torque than type-I fibres because of a greater amount of Ca^2+^ released per action potential, faster muscle proteins isoforms and cross-bridge formation rates (Maffiuletti et al. 2016). This had implications on the skeletal muscle capacity to produce rapid torque (Harridge et al. 1996). On the other hand, type-II fibres are more sensitive to muscle damage as compared to type-I fibres (Lieber and Friden 1988). Indeed, greater muscle damage was observed when comparing the same relative eccentric exercise performed by elbow flexors vs leg extensors (Jamurtas et al. 2005), with the former showing greater type-II fibre prevalence than the latter (Saltin and Gollnick 2011). To our knowledge, the only study that has previously assessed RVFD following a downhill running bout did not distinguish the early from the late phase, while it reported the force exerted at 30 ms, 50 ms, 100 ms and 200 ms (Maeo et al. 2017). Notwithstanding, this was investigated in both the knee extensors and the plantar flexors, opening for interesting comparisons (Maeo et al. 2017). For instance, the authors observed that the force exerted at 200 ms was impaired in the knee extensors but not in the plantar flexors, in line with the possible relationship between the type-II prevalence and the late phase (Maeo et al. 2017). However, the authors also found impairments in the force exerted at 50 ms (Maeo et al. 2017), which contrasts with what we observed in the present study. However, besides the different downhill running protocols [45 min at −15% (Maeo et al. 2017) vs 30 min at −20% in the present study), our participants were recreational runners, while the others (Maeo et al. 2017) were described as young adults with no specific experience in running. Since the extent of muscle damage is greater in untrained vs trained individuals (Newton et al. 2008), it is possible that those participants may have experienced greater muscle damage, also affecting the neural aspects as already seen after eccentric exercise (Prasartwuth et al. 2005). Summarizing, the muscle damage following the downhill running bout may have affected the muscle structure and especially the type-II fibres, whose characteristics are important determinants of the RVFD late phase (Peñailillo et al. 2015).

The exposure to downhill running is followed by alterations in muscle damage markers. We observed decrements in knee extensors MVIC that recovered after 4 days, coupled with increments in CK blood concentration, muscle swelling and delayed soreness. The decrement in maximum force derives from both neural and structural factors, while the increment in CK blood concentration is an index of the rupture of the myonuclei, and the increment in muscle swelling and soreness is mainly due to the inflammation process following the eccentric exercise (Proske and Morgan 2001).

### Limitations

First, the present results refer to downhill running, and other eccentric exercise modalities may produce different effects. Second, different durations and/or slopes, as well as different populations may result in dissimilar outcomes. Third, the sample size was small and should be increased for more definitive conclusions. Fourth, further muscle damage markers such as the echo-intensity or the shift in optimum angle could have deepened the discussion. Last, we only investigated the knee extensors, and different muscles in combination with different eccentric exercises may differ in behaviour.

## Conclusions

A downhill running bout impaired the late but not the early phase of the knee extensors RVFD for the following 72 h, with concomitant decrements in knee extensors maximum force and increments in serum CK concentration, muscle swelling and soreness. Overall, all muscle damage markers recovered in 4 days. The rate of voluntary force development, and especially its late phase, appears sensitive to muscle damage and could be used as an additional muscle damage marker in trail running and following prolonged duration races wherein cumulative eccentric loading is high.

## Data Availability

Derived data supporting the findings of this study are available on request from the corresponding author.

## Abbreviations

CK: Creatine kinase
MVIC: Maximum voluntary isometric contraction
POST24: Neuromuscular evaluation performed 24 h after the fatiguing exercise
POST48: Neuromuscular evaluation performed 48 h after the fatiguing exercise
POST72: Neuromuscular evaluation performed 72 h after the fatiguing exercise
POST96: Neuromuscular evaluation performed 96 h after the fatiguing exercise
PRE: Neuromuscular evaluation performed before the fatiguing exercise
RVFD: Rate of voluntary force development
RVFD_0–50_: Rate of voluntary force development calculated at the 0–50 ms time interval
RVFD_100–200_: Rate of voluntary force development calculated at the 100–200 ms time interval
